# A Simple Method of Electrospun Tungsten Trioxide Nanofibers with Enhanced Visible-Light Photocatalytic Activity

**DOI:** 10.1007/s40820-015-0042-8

**Published:** 2015-06-04

**Authors:** Frank Agyemang Ofori, Faheem A. Sheikh, Richard Appiah-Ntiamoah, Xinsheng Yang, Hern Kim

**Affiliations:** 1 Department of Energy Science and Technology, Energy and Environment Fusion Technology Center, Myongji University, Gyeonggi-do Yongin, 449-728 Republic of Korea; 2 Key Laboratory of Advanced Technology of Materials, Superconductor and New Energy R&D Center, Southwest Jiaotong University, Chengdu, 610031 People’s Republic of China

**Keywords:** Tungsten trioxide, Electrospinning, Nanofiber, Photocatalysis

## Abstract

The present study involves the fabrication of tungsten trioxide (WO_3_) nanofibers by an electrospinning technique using polyvinyl pyrrolidone (PVP)/citric acid/tungstic acid as precursor solution. It was found that the PVP concentration was one of the most crucial processing parameters determining the final properties of WO_3_ nanofibers. The optimum concentration of PVP was from 75 to 94 g L^−1^. The average diameter of the nanofibers increases with increasing the PVP concentration, whereas it is decreased after sintering and orthorhombic structure were formed at 500 °C. The photocatalytic properties of the as-synthesized nanofibers were also investigated by degrading methylene blue and twofold efficiency was obtained compared with that of commercial WO_3_ microparticles.

## Introduction

The textile industry effluent is a major source of water pollution which can destroy the aquatic life and influence world climates change seriously [1]. The main source of water pollution comes from dyeing and finishing process of fabrics in textile industries. It is believed that 10–15 % of the dyes are unintentionally wasted during the dyeing processes in major developing countries [2]. An industrial dye constitutes one of the largest groups of harmful organic compounds which are difficult to degrade naturally. Mostly, the non-fixed dyes especially the azo-dyes and other inorganic salts from textile factories tend to contain considerable amount of toxins and are highly carcinogenic. Therefore, direct contact of these dyes has harmful effects on human [3, 4].

Currently, different strategies have been employed for treating the textile effluents before they are released in water bodies including microfiltration [5], chemical precipitation [6], biosorption [7], membrane separation techniques [8], nanofiltration [9], electrochemical treatment [10] solvent extraction [11], and coagulation-flocculation [12]. However, the limitations of these strategies include that incomplete ion removal and production of toxic sludge are unavoidable, which require further disposal of the released effluents. The residual dyes can remain for a longer period of time which are non-biodegradable and have no effect due to sunlight [13]. Hence, the aforementioned methods are not fully useful as they can only transform the dyes from one phase to another, creating new kinds of pollutants which further need to be treated with other methods such as advanced oxidation processes by photocatalytic degradation using metal oxides.

Tungsten trioxide (WO_3_) is one of the most popular metal oxides which have been extensively used as electrochromic devices and gas sensor for water splitting [14]. Recently, it was concluded that WO_3_ has a broad range of band-gap values which ensures considerable photocatalytic and photoelectrocatalytic activity under visible-light illumination [15]. At this point of view, WO_3_-based materials as well as their photocatalysis have attracted much attention, for example, nanoparticles for degradation of lidocaine under visible and sunlight irradiation [16] and porous structure for degradation of rhodamine B dye [17]. Many efforts have also been made to enhance the photocatalytic efficiency of WO_3_, for example, photo-deposition of platinum in WO_3_ nanostructures [18], titania-WO_3_ nanotubular composite fabricated by electrochemical method [19], various films based on WO_3_ [20], etc. However, WO_3_ nanofibers with large aspect ratio are rarely reported before [21, 22]. It is believed that increase of surface-to-volume ratio is one of the effective ways to increase the photocatalytic efficiency [23]. In this connection, various nano-structured WO_3_ such as nanorods [24], nanofibers [25], nanowires [26], and nanoporous films [27] have been fabricated. Among these nanomaterials, nanofibers with superior porosity, excellent mechanical properties, and desirable chemical properties make them multifunctional capabilities for various applications [28].

Generally, nanofibers are produced by three techniques of self-assembly [29], phase separation [30], and electrospinning [31], in which the electrospinning is considered to be the most preferred technique due to its facility of quantity production, tunable properties via changing the solution parameters, as well as wide applications. Till now, polymer, ceramic, metal oxide, and lots of composite nanofibers have been successfully prepared via electrospinning technique [31–34]. Various electrospun WO_3_-based nanofibers were also investigated widely, for example, WO_3_ nanofibers for gas-sensing [35], core–shell WO_3_/TiO_2_ nanofibers for photocatalysis [36], polycrystalline trioxide nanofibers as ammonia sensors [37], templates for detection of biomarker molecules [38], and improved photoluminescence [25]. Especially, WO_3_ nanofibers can be efficiently used for photocatalyst due to its unique physical and chemical properties as above references mentioned. Recently, lots of works have been done to investigate the fabrication and properties of electrospun WO_3_ nanofibers [25, 26, 37, 39]. However, the existing methods need either expensive precursors or long time process. In this work, we developed a simple electrospun method of fabrication WO_3_ nanofibers by using inexpensive precursor of polyvinylpyrrolidone (PVP)/citric acid/tungstic acid. The photocatalytic efficiency of the as-synthesized nanofibers was also investigated via degrading methylene blue.

## Experimental

### Materials

PVP of *M*_w_ = 1,300,000 was purchased from Sigma Aldrich in USA. Commercial WO_3_ powders (mean particle size 30 μm) were obtained from Kanto Chemical Company and methylene blue (99 % purity) from Showa Chemical Company in Japan. Ammonia solution was from Duskan Pure Chemical Company Ltd, and citric acid monohydrate (99.5 % purity) was from Samchun Pure Chemicals in Republic of Korea.

### Preparation of Solution for Electrospinning

The WO_3_ nanofibers were synthesized using an electrospinning method. Commercially available WO_3_ powders were dissolved in hot ammonia solution with stirring firstly. After the mixture was cooled down to room temperature, the insoluble materials were removed by filtration after which an amount of citric acid was added as chelating agent for binding WO_3_ [40]. Then, PVP (75 g L^−1^) were added into the above solution followed by magnetic stirring for about 1 day. The weight ratio of PVP/citric acid/WO_3_ was kept constant at a ratio of (1.1:2.7:1.0). The mixed solution was aged on hot plate for different time to form different concentration of PVP/citric acid/tungstic acid precursor gel for electrospinning. In this work, three kinds of precursor solution were prepared in which the concentration of PVP was 75, 94, and 140 g L^−1^, respectively.

### Electrospinning Solution

A high voltage power supply (CPS-60 K02V1, Chungpa EMT Co.in Republic of Korea) capable of generating voltages up to 30 kV was used as the electric field source for spinning of nanofibers. Solutions to be electrospun were supplied through a 20-mL plastic syringe attached with stainless steel having 22 gages, (0.7 mm OD × 0.4 mm ID). The feeding rate of solution to be electrospun was adjusted to be 0.2 mL min^−1^ by using a syringe pump (KDS-100, KD Scientific, United States). The syringe was aligned perpendicular to flat bed collector of electrospinning apparatus. The wire originated from the positive electrode (anode) was connected with the needle tip of plastic syringe with an alligator clip. Finally, the solutions were electrospun at +18 kV. The distance between the needle tip and the flat bed metallic collector covered with aluminum foil was kept at 15 cm. The as-spun nanofibers were vacuously dried at 60 °C for 24 h in the presence of P_2_O_5_ to remove the residual moisture from used solvents. Further on, the samples were subjected to calcination at 500 °C for 2 h with a heating rate of 1 °C min^−1^.

### Characterization

The morphology of the samples was examined using a scanning electron microscope (SEM, Hitachi, Japan S-3500N). The samples were coated with a thin layer of platinum–palladium for 50 s with the discharge current of 10–15 mA for three reparative cycles using (Hitachi, Japan E1010) ion sputter. After coating, the micrographs were taken at an accelerating voltage of 25 kV, and images were captured with magnifications of 1 and 20 K. The thermal properties were tested by thermogravimetry/differential scanning calorimetry instrument (SETARAM TG/DSC 92). The samples were heated from 30 to 600 °C under a continuous nitrogen purge of 20 mL min^−1^ with a heating rate of 20 °C min^−1^. Information about the phases and crystallinity of nanofibers was obtained using X-ray diffractometer (XRD, X’pert-pro MPD) with Cu, Cr (*λ* = 1.540 A) radiation over Bragg angle ranging from 20° to 60°. For photocatalytic property, the samples were suspended (1 g L^−1^) in methylene blue solution (20 ppm) in a batch reactor and illumination was applied using a commercial haloline visible-light illumination (500 W, Osram). The evolution of the methylene blue concentration was measured by UV–Vis spectrometry (Cary 100, Varian) following its 660 nm characteristic band.

## Results and Discussion

**Fig. 1:**
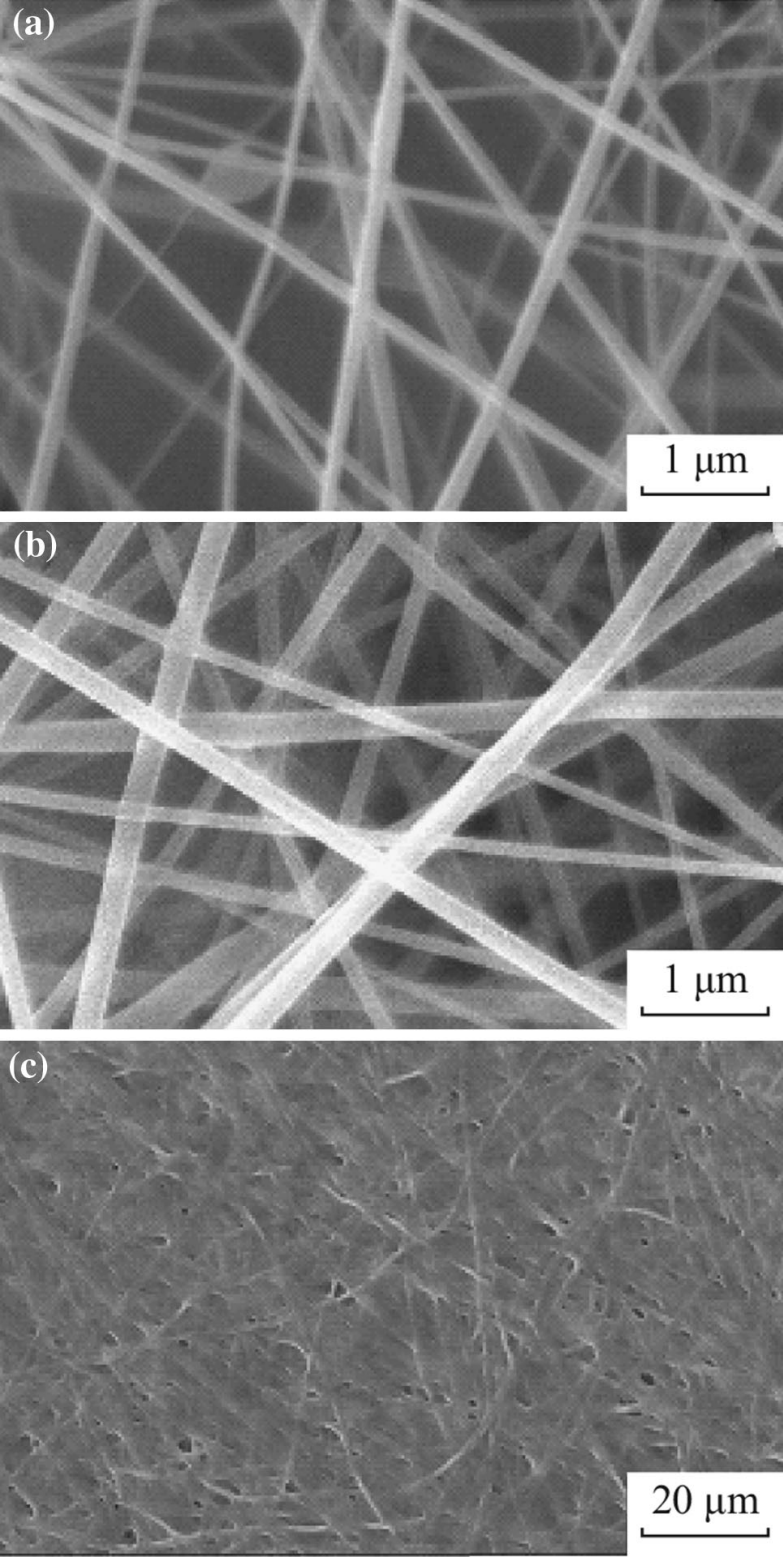
SEM images of the as-spun nanofibers for different PVP concentrations of 75 g L^−1^ (**a**), 94 g L^−1^ (**b**), and 140 g L^−1^ (**c**)

**Fig. 2:**
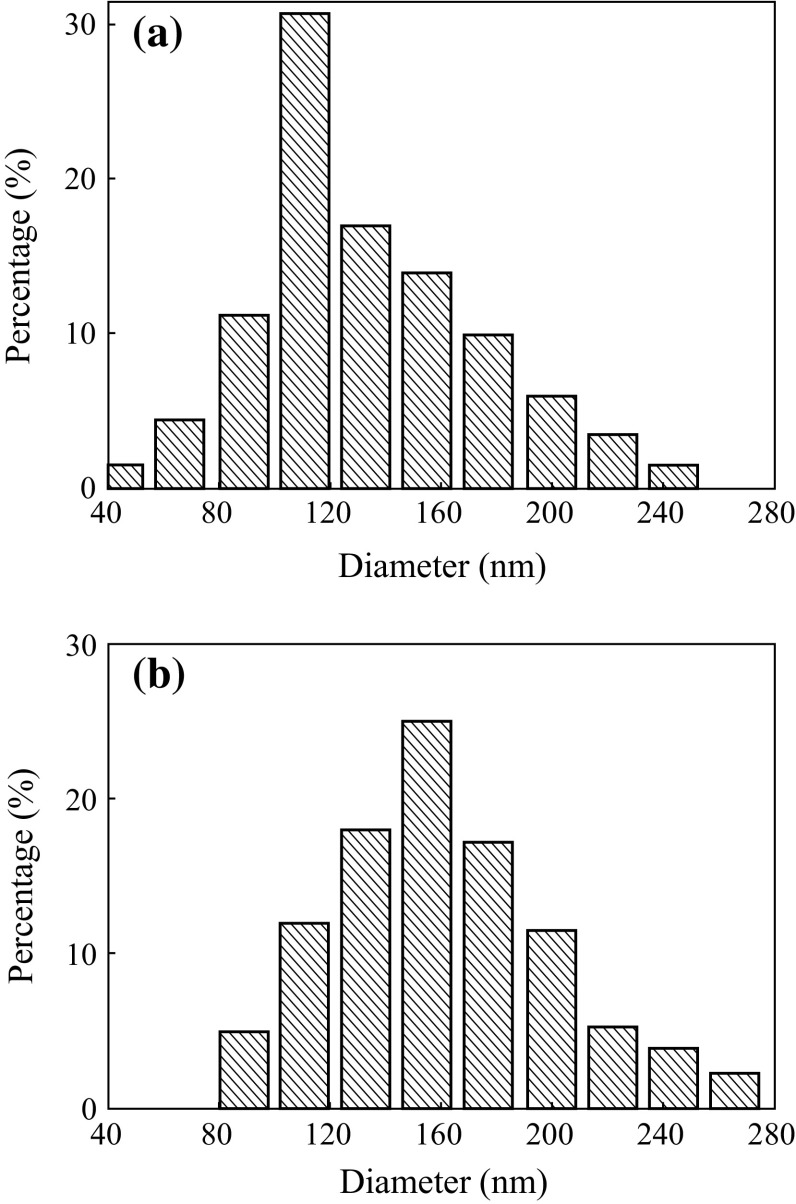
Size distributions of the as-spun nanofibers for different PVP concentrations of 75 g L^−1^ (**a**) and 94 g L^−1^ (**b**)

Figure 1 shows the SEM images of the as-electrospun nanofibers at different PVP concentrations. It was observed that the PVP concentration strongly influenced the ability of spinning, in terms of fiber formation and fiber diameters. For instance, at lower PVP concentration (i.e., less than 75 g L^−1^), electrospinning process could not be carried out due to low viscosity of precursor solutions (data not shown). However, when PVP concentration was 75 g L^−1^ or higher, the electrospinning was highly achievable for nanofibers. Figure 1a–c shows, respectively, the results of 75, 94, and 140 g L^−1^ of PVP. From Fig. 1a, b, one can see that nanofibers are bead and defect free without conglomeration, whereas they are conglomeration when fabricated at 140 g L^−1^ (Fig. 1c). Figure 2 shows the size distribution of nanofibers calculated by randomly selecting 30 nanofibers from SEM images. The diameters are in the range of 40–280 nm when PVP concentration is 75 and 94 g L^−1^, and the average diameter of the nanofibers increased with PVP concentrations. These results are in agreement well with the conclusion made by Yan et al. [41].

**Fig. 3:**
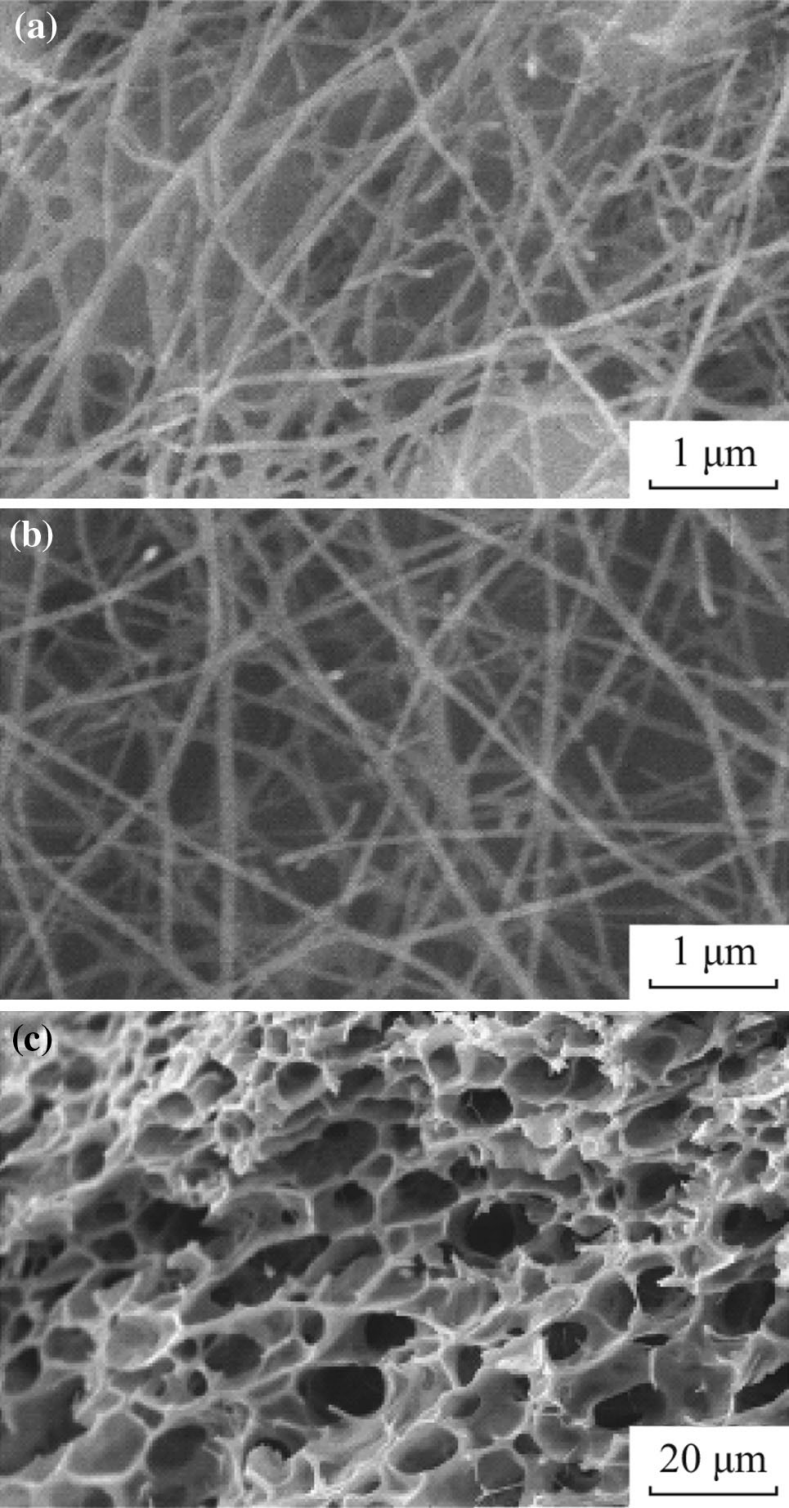
SEM images of nanofibers calcined at 500 °C for different PVP concentrations of 75 g L^−1^ (**a**), 94 g L^−1^ (**b**) and 140 g L^−1^ (**c**)

**Fig. 4:**
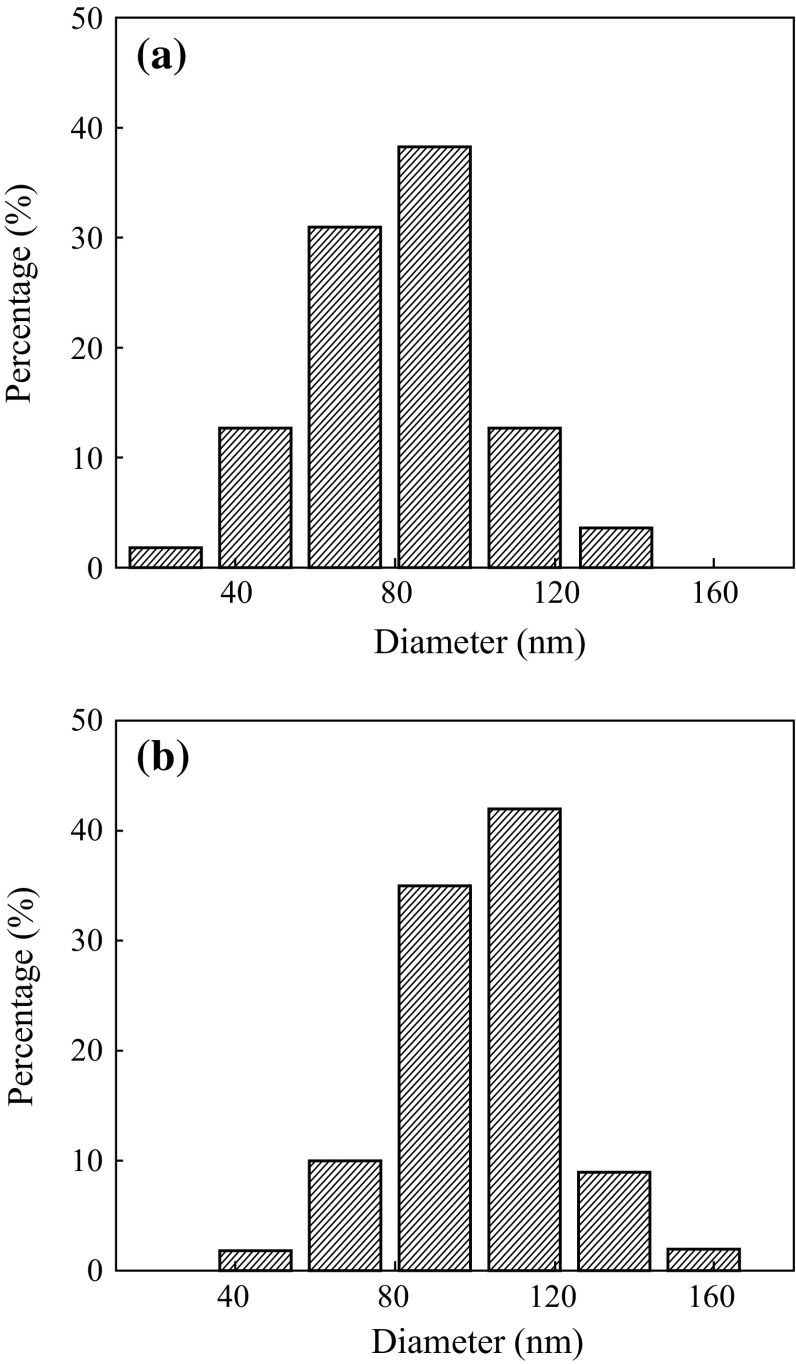
Size distributions of nanofibers calcined at 500 °C for different PVP concentrations of 75 g L^−1^ (**a**) and 94 g L^−1^ (**b**)

The SEM images of the nanofibers calcined at 500 °C for different PVP concentration are shown in Fig. 3. It can be seen that the nanofibers obtained at PVP concentration of 75 and 94 g L^−1^ maintained good fibrous morphology (Fig. 3a, b), whereas the nanofibrous morphology fabricated at 140 g L^−1^ of PVP concentration was completely distorted and porous nest-like structure was formed. The size of nanofibers after calcinations was reduced to 80–100 nm for 75 and 94 g L^−1^ PVP concentrations due to the removal the polymer precursor. The diameter distribution in the range of 20–160 nm is shown in Fig. 4a, b, which is comparatively less than the as-spun nanofibers. This may be indicated that the PVP precursor was removed from the nanofibers after calcination and pure metal oxide nanofibers were yielded.

**Fig. 5:**
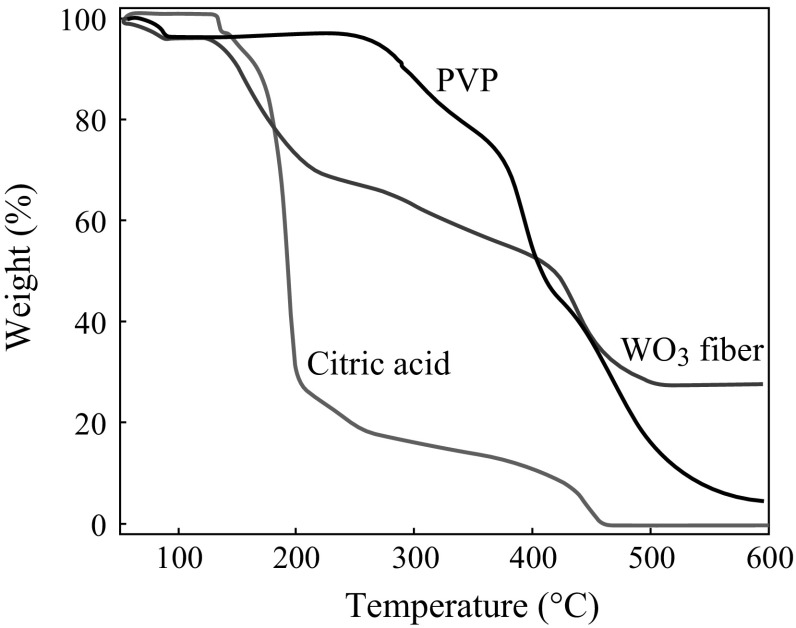
TGA curves of nanofibers at 500 °C, pure citric acid, and PVP

**Fig. 6:**
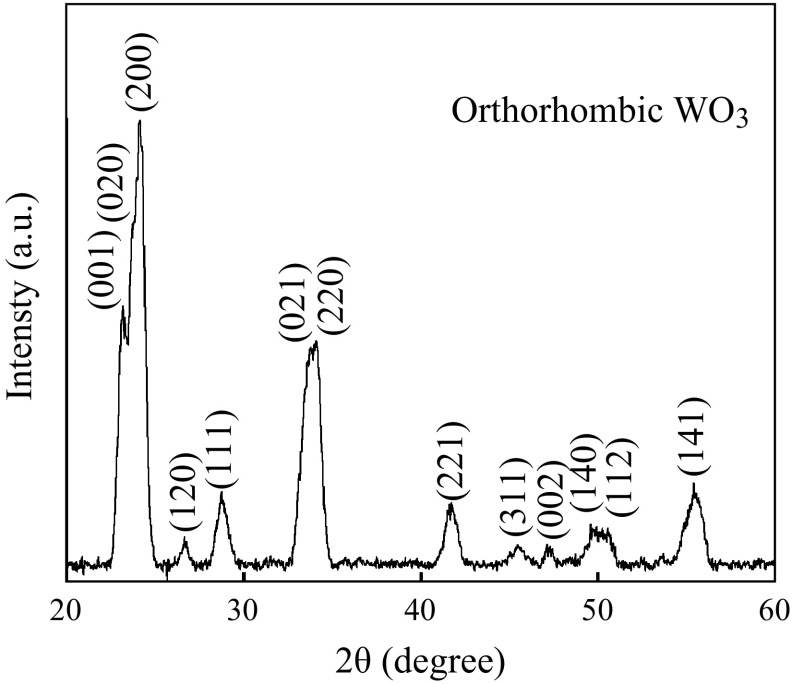
XRD pattern of nanofibers fabricated at 75 g L^−1^ PVP concentration and calcined at 500 °C

In order to confirm the precursor was completely removed after calcination, TGA of pure PVP, citric acid, and WO_3_ nanofibers were performed. The results of WO_3_ nanofibers indicate that residual weight is about 25 % at 500 °C, while it is 13 % for pure PVP and no citric acid remained (see Fig. 5). This indicates that the polymer in the as-spun nanofibers can be easily removed by sintering the sample at 500 °C. The typical XRD pattern of WO_3_ nanofibers is presented in Fig. 6. The diffraction peaks agree well with the standard orthorhombic WO_3_ crystal (JCDPS, card no 05-03888).

**Fig. 7:**
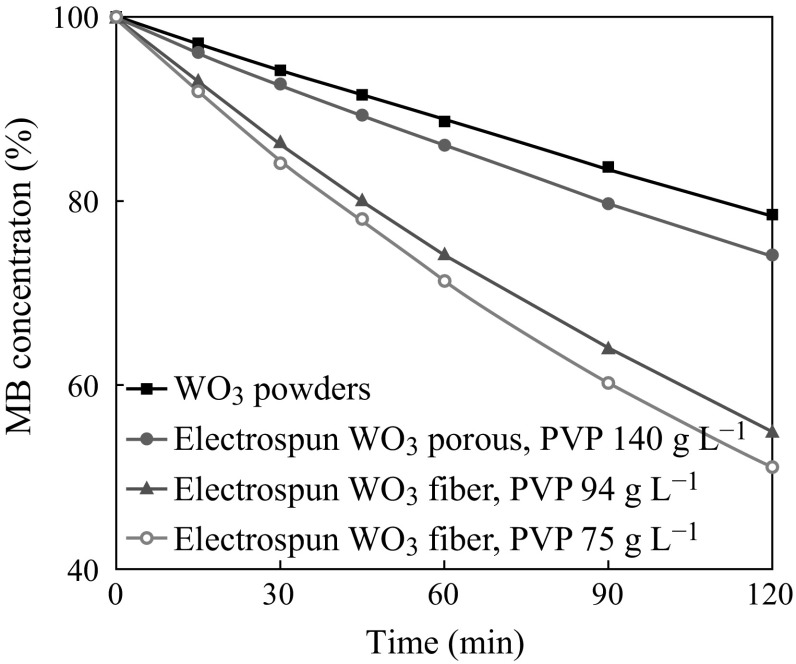
Photocatalytic degradation of commercial WO_3_ powders, WO_3_ nanofibers fabricated from 75 g L^−1^, 94 g L^−1^, and 140 g L^−1^ PVP precursor under visible-light illumination

Figure 7 shows the photocatalytic result of WO_3_ nanofibers by degrading methylene blue under visible-light illumination. For comparison, the commercial powders (mean particle size of 30 μm) were also investigated simultaneously. After irradiation for 2 h, the degradation efficiency for commercially available WO_3_ powders was only about 23 %, while the degradation efficiency for WO_3_ nanofibers (PVP: 140 g L^−1^) was 27 %, (PVP: 94 g L^−1^) 46 %, and (PVP: 75 g L^−1^) 50 %, respectively. The degradation efficiency for nanofibers is much higher than that of commercially available WO_3_ particles of which mean diameter is 30 μm according to manufacturer. It can be observed that the degradation efficiency for nanofibers with the smaller diameter prepared from (PVP: 75 g L^−1^) was 50 % which is higher than other nanofibers combinations with lager diameter. Therefore, it can be concluded that electrospinning WO_3_ nanofibers with smaller diameters can significantly improve the photocatalytic activity than commercially available WO_3_ micro-powders.

## Conclusion

WO_3_ nanofibers were fabricated by an electrospinning technique using PVP/citric acid/tungstic acid as precursor solutions. The average diameter of nanofibers is 80–100 nm and increases with PVP concentration. After sintering at 500 °C for 2 h, the diameter was reduced and the structure was transferred from amorphous to orthorhombic. The photocatalytic activity of WO_3_ nanofibers was found much higher than that of commercial WO_3_ powders in degradation of methylene blue under visible-light illumination.
